# Cell mediated immunity against HPV16 E2, E6 and E7 peptides in women with incident CIN and in constantly HPV-negative women followed-up for 10-years

**DOI:** 10.1186/s12967-015-0498-9

**Published:** 2015-05-20

**Authors:** Anna Paaso, Hanna-Mari Koskimaa, Marij JP Welters, Seija Grénman, Kari Syrjänen, Sjoerd H van der Burg, Stina Syrjänen

**Affiliations:** Medicity Research Laboratory and Department of Oral Pathology, Institute of Dentistry, Faculty of Medicine, University of Turku, Turku, Finland; Department of Obstetrics and Gynaecology, Turku University Hospital, Turku, Finland; Department of Clinical Research, Biohit Oyj, Helsinki, Finland; Department of Clinical Oncology, Leiden University Medical Center, Leiden, The Netherlands; Department of Oral Pathology, Institute of Dentistry, Faculty of Medicine, University of Turku, Lemminkäisenkatu 2, FI 20540 Turku, Finland

**Keywords:** Human papillomavirus type 16, Cell-mediated immunity, Clearance of HPV infection, Cervical intraepithelial neoplasia, Common antigen, LST

## Abstract

**Background:**

Virus-specific cell-mediated immunity (CMI) plays a role in the outcome of genital HPV infections. To cast further light on the question why most women clear their HPV infection while others develop high-grade cervical intraepithelial neoplasia (CIN), we analyzed HPV16 E2-, E6- and E7 -specific CMI in women who developed CIN during a 10-year follow-up of the Finnish Family HPV cohort.

**Methods:**

Overlapping 30–35 mer peptides covering the entire HPV16 E2-, E6- and E7 protein sequences were used for defining the lymphocyte proliferation capacity, cytokine production (IL-2, IL-5, IL-10, IL-17A, IFN-γ and TNF-α) and numbers of HPV16 -specific CD4+ CD25+ Foxp3+ regulatory T-cells in 10 women who developed CIN, and in 22 control women who tested constantly HPV-negative during the follow-up. HPV-specific CMI was related to the demographic data including sexual behavior, smoking and alcohol consumption.

**Results:**

Women with CIN and their controls had similar T-cell mediated immunity against HPV16 E2, E6 and E7 peptide pools. However, nearly fourfold higher T-cell reactivity against common antigens was found in the CIN women than in the healthy donors (p = 0.001). HPV16 E6 stimulation resulted in higher IL-17A secretion in the controls than in the CIN women (p = 0.035). Smoking and use of alcohol affected the T-cell response to common antigens but not to HPV peptides (p = 0.032 and 0.045, respectively).

**Conclusion:**

While both the CIN women and controls exhibited an HPV16-specific CMI, IL-17A might be of importance in HPV induced pathology. The hyper-responsiveness of the CIN patients to common antigens needs further studies. Smoking and alcohol had no effect on HPV-specific CMI.

**Electronic supplementary material:**

The online version of this article (doi:10.1186/s12967-015-0498-9) contains supplementary material, which is available to authorized users.

## Background

Currently, nearly 200 different human papillomavirus (HPV) types have been identified infecting either skin or mucosa. The mucosal HPVs are classified as either high- (HR) or low-risk (LR) types based on their oncogenic potential [1]. The most common HR-type is the HPV16 followed by HPV18, associated with pre-cancers and cancers in different anogenital sites, including uterine cervix, vagina, vulva, and anus [2,3]. HPV16 is the most common genotype also in asymptomatic infections of the head and neck region and may cause cancers also there [4,5]. Persistent genital HPV infections have been associated with impaired HPV-specific CMI, whereas CD4+ and CD8+ HPV-specific T-cell response might result in spontaneous regression of cervical disease [6], and with a better survival of the patients with cervical cancer [7]. Also vaccine-induced HPV-specific T-cells play an active role in tumor regression [8,9]. Most studies on the spontaneous HPV-specific responses have been performed in cohorts were the previous HPV-DNA and serological status of the participating women have been unknown at study entry. Thus, it is difficult to estimate at which stage the women have developed HPV-specific T-cell responses [7,8,10-12].

The majority of healthy women without any genital HPV disease mount blood T-cell responses against HPV16 E2, E6 and E7 proteins [12,13]. In patients with persistent or progressive infections, the T-cell response to HPV16 E2 peptides might be a better indicator of viral clearance than T-cell response to HPV16 E6 and/or E7 [14,15]. Integration of HPV16 results in disruption of E2 and up-regulation of E6 and E7 expression [14,16]. Given that E2 protein is absent in viral particles but needed in viral replication, E2 T-cell responses might indicate a replicative HPV infection [17]. Recent experimental evidence suggests that HPV16 E2 has an anti-tumor effect by promoting innate immunity and apoptosis [18,19]. Thus, CMI against E2 protein could be the most likely candidate to reflect differences between the women with and without cervical diseases.

In the present study, we analyzed the HPV16 E2-, E6- and E7-specific CMI both in women (n = 10) who developed CIN and in those who remained disease free (n = 22) during the entire 10-year follow-up (FU) as participants in the Finnish Family HPV Study. In addition to the genital HPV status, also the oral HPV DNA status and HPV serostatus were known during the FU.

## Methods

### Cohort

The Finnish Family HPV Study is a longitudinal cohort study conducted at the Department of Oral Pathology, Institute of Dentistry, University of Turku and Department of Obstetrics and Gynecology, Turku University Central Hospital. Originally, 329 pregnant women at their 3rd trimester pregnancy and all their newborns (n = 331; includes two twins) were enrolled in the study between 1998 and 2001, as described previously [20,21]. The study plan was approved by the Research Ethics Committee of Turku University Hospital (O66/10, TO7/008/14). During the FU until present, 12 women developed an incident CIN of which 10 women participated in this study. Five women developed CIN3, three CIN2 and two CIN1. In total 5 of the 10 biopsy samples taken from CIN lesions at the time of diagnosis and/or treatment tested HPV16 positive. All CIN2 and CIN3 lesions were treated with conization and followed by Pap tests according to the routine [22]. The time point of the treatment is given in Additional file 1: Table S1. A nested, control group for the CIN cases was selected comprising 22 women who remained cervical disease free during the 10-year FU. Written informed consent was obtained from all 32 women. At the blood sampling for CMI studies, the mean age of the CIN women and their controls was 37 years and 40 years, respectively.

### HPV testing during the FU

The natural history of genital and oral HPV infection in women participated in the Finnish Family HPV Study has been published earlier [4,5,20,23,24], as well as the dynamics of HPV serology to the L1 major capsid protein of HPV 6, 11, 16, 18 and 45 [25]. The methods used in these previous studies are described shortly as follows:

### Oral and cervical samples

Oral samples for HPV testing were taken during the FU at the baseline and at months 2, 6, 12, 24, 36 and 6.5 years. Samples were taken from the buccal mucosa of both cheeks and upper and lower vestibular areas with a brush (Cytobrush®, MedScan, Malmö, Sweden) into 80% ethanol and were immediately frozen and stored at -70°C until use. At the last visit, a dentist also performed a careful clinical examination of the oral mucosa. The cervical scrapings for HPV testing were taken with a brush (Cytobrush, Medscand, Malmö, Sweden) at the same time points as the oral samples during the 10 years FU. The brushes were placed in a tube with 0.05 phosphate-buffered saline (PBS) with 100 μg of gentamycin and immediately frozen at -20°C and the stored at -70°C until used [20].

### HPV testing

HPV DNA was extracted from the scrapings with the high salt method [26]. HPV testing was performed with nested PCR using My09/My11 and GP05+/GP06+ primers. HPV genotyping was performed with a multiplex HPV genotyping kit (Multimetrix; Progen Biotechnik GmbH) which identifies 24 LR- and HR-HPV-genotypes: LR-HPV: 6, 11, 42, 43, 44 and 70 and HR-HPV: 16, 18, 26, 31, 33, 35, 39, 45, 51, 52, 53, 56, 58, 59, 66, 68, 73 and 82 [27].

### HPV serology

Blood samples for antibody determination were taken at baseline and at 12, 24 and 36 months of the follow-up. Samples were centrifuged at 2400 rpm for 10 min (Sorval GLC-2; DuPont instrument) and the serum was divided into three 1 ml aliquots and stored at -20°C for no longer than one week and then at -70°C until sent for analysis at the DKFZ, Heidelberg, Germany [28]. Antibodies to the major capsid protein L1 of HPV types 6, 11, 16, 18 and 45 were analyzed by multiplex HPV serology based on glutathione S-transferase fusion-protein capture on fluorescent beads, as described earlier [29].

### Demographic data collected by questionnaire

At study onset and on the last visit, all women filled in a standardized questionnaire recording data on their socioeconomic status, general health, sexual and reproductive behavior, smoking and alcohol consumption, medication and history of sexually transmitted disease (STDs) [20,24]. Altogether, 36 items were included in the questionnaire.

### Blood samples for CMI

The isolation of peripheral blood mononuclear cells (PBMCs) has been described earlier [30]. Briefly, venous blood samples (74 mL) were collected in sodium-heparin collection tubes and the isolation was done by centrifugation over Ficoll-Paque gradient (GE Healthcare Life Sciences, Uppsala, Sweden). PBMCs were subjected to lymphocyte stimulation test (LST) at the concentration ~10x10^6^ and the leftover cells were frozen in 80% Fetal Bovine Serum (FBS, Biowest) and 20% DMSO (Merck, Darmstadt, Germany). Autologous serum obtained from a clotting tube was used as serum in the short-term T-cell proliferation assay as described previously [10,30,31].

### HPV16 peptides

Collection of overlapping 30-35 mer peptides with HPV16 E2, E6, and E7 protein sequences were synthesized by solid phase peptide synthesis (SPPS) method with >95% purity (ChinaPeptides Co. Shanghai, China) with a 14 (for 30-mer) or 15 (for 35-mer) amino acid (aa) overlap as described previously [30]. There were two pools of E2 peptides (E2.1 and E2.2) which consisted of 12 or 11 (30-mer) peptides, respectively. Four pools of E6 and two pools of E7 peptides (E6.1-E6.4 and E7.1 and E7.2) consisted of two 32-mer or 35-mer peptides, respectively. Memory response mix (MRM) stock solution (50x), consisted of tetanus toxoid, 0.75 fL/mL (Statens Serum Institut, Copenhagen, Denmark), Tuberculin PPD, 5 μg/mL (Statens Serum Institut), and *Candida albicans*, 0.015% (Greer Laboratories, Lenoir, USA) and it was used as a positive control for the proliferation assays and cytokine production capacity of the PBMCs [8,13,30].

### HPV16–specific T-cell proliferative capacity by LST

The protocol for LST is described in details recently [30]. The PBMCs were seeded into U-bottomed 96-wells microtiter plate (Nunc, Roskilde, Denmark) with density of 1.5×10^5^/well and each peptide pool were culture with eight replicative wells. IMDM (Gibco, Life Technologies, Belgium) was used as a cell culture medium with 10% autologous serum. Each peptide pools were used at a final concentration of 5 μg/mL. PBMCs cultured with MRM were used as a positive control (10 μl/well of 4xMRM) and with no antigen (medium-only) as a background control. After 6 days of culturing the supernatants of all eight replicative wells were collected and pooled for cytokine analysis. A compensatory amount of IMDM supplied with 0.5 μCi [^3^H]-Thymidine (PerkinElmer, Turku, Finland) per well was added. After 16-18 hours of incubation, the cells were harvested into Unifilter plates (PerkinElmer) using the FilterMateTM Cell Harvester (PerkinElmer). Subsequently, the filter plates were dried and counted on the 1450 MicroBeta + counter (PerkinElmer). The cut-off value for counts per minute (CPM) values was determined by the average plus 3 × SD of the eight medium-only control wells. Stimulation index (SI) was calculated as the average of tested eight wells divided by the average of the medium-only control wells. The proliferative response was defined positive if the CPM values of at least six of the eight wells were above the cut-off value and if the SI was ≥3 [12,13].

### Cytokine polarization analysis

The levels of IFN-γ, TNF-α, IL-2, IL-5, IL-10, and IL-17A were determined from supernatants collected from LST at day 6 with the Cytometric Bead Array (CBA) human enhanced sensitivity flex set system (BD Biosciences, Temse, Belgium) as described previous [30]. The detection limits for the cytokines were based on standard curves complying with the limit of 274 fg/mL described by the manufacturer. The limit of positive antigen-induced cytokine production was determined as a cytokine concentration > 2× the concentration of the medium-only control [7].

### HPV16 –specific CD4 + CD25 + Foxp3+ regulatory T-cells

Thawed PBMCs were seeded into 24-wells plate at the density of 1.0 × 10^6^ cells/well and the cells were cultured in IMDM containing 10% Human AB serum (Sigma-Aldrich, San Louis, USA). More detailed protocol is written in our previous manuscript [30]. Cells were stimulated with HPV16 E6 or E7 peptide pools or cultured in medium only and at day 7 they were stained first with surface markers CD25 diluted 1:25, stock concentration 10 μg/mL (Anti-CD25-FITC, clone 2A3, BD Pharmingen, San Jose, CA); CD4, diluted 1:100, no information on stock concentration was provided by manufacturer (Anti-CD4-APC, clone RPA-T4, BD Pharmingen); CD8, diluted 1:30, stock concentration 5 μg/mL (Anti-CD8 PerCP-Cy5.5; clone SK1, BD Pharmingen). Subsequently, the cells were fixed and permeabilized using intra-nuclear staining buffer set (FOXP3 Fix/Perm buffer set, Biolegend, San Diego, CA) according to manufacturer’s instructions. An antigen-induced alteration in the population percentage was defined as a change of at least 2× the corresponding percentage in the medium-only control [7,31].

### Statistical analysis

All statistical analyses were run using IBM SPSS® (IBM, Inc., New York, USA) software package (IBM SPSS Statistics for Windows, version 22.0.0.1). Frequency tables were analyzed using the *χ*2-test, with the likelihood ratio or Fisher’s exact test for categorical variables. Differences in the means of continuous variables were tested using non-parametric (Mann-Whitney) tests for two independent samples. Bonferroni correction was used in ANOVA with multiple comparisons. All statistical tests were two-sided and considered significant at p-value ≤0.05.

## Results

### HPV status during the FU

Additional file 1: Table S1 summarizes the HPV status of the 10 women with incident CIN and the 22 HPV-negative controls. Nine women who developed CIN had HPV16 detectable in her cervix at some time point during the FU. Eight of these women tested HPV16-positive several times during the FU which indicates persistent HPV16 infection. Of these possible HPV16 persistors, three had no serum antibodies against HPV16 in any of the four serial samples during the FU. Oral samples tested HPV16 DNA positive in four CIN patients of whom three had HPV16 DNA detectable also in the cervix. HPV16 specific antibodies were found in three of the four women with oral HPV. Of the 22 control women, eight had HPV DNA detectable in the oral mucosa, of which only two were HPV16-seropositive.

### HPV16 E2-, E6- and E7-specific proliferative T-cell response

**Figure 1:**
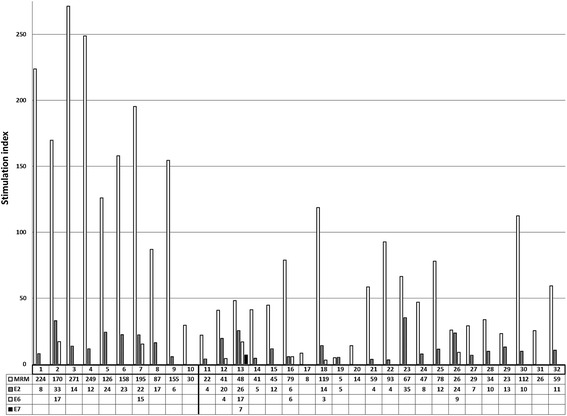
LST results of the two study groups. The results of lymphocyte stimulation test (LST) of women with CIN (IDs 1-10) and healthy controls (IDs 11-32) after stimulation with HPV 16 E2, E6 and E7. The stimulation indexes (positive responses) of peptide pools E2, E6 and E7 are combined and the mean values for each woman are presented in the table. MRM = memory response mix.

Figure 1 summarizes the results on HPV16-specific T-cell immunity against E2, E6 and E7 in the CIN women and their controls. HPV16 E2.1 and E2.2 peptide pool specific reactivity was found in 8/10 (80%) and 5/10 (50%) cases and in 15/22 (68%) and 14/22 (64%) controls, respectively. No statistically significant difference in reactivity against these pools between the two groups (p > 0.05).

**Figure 2:**
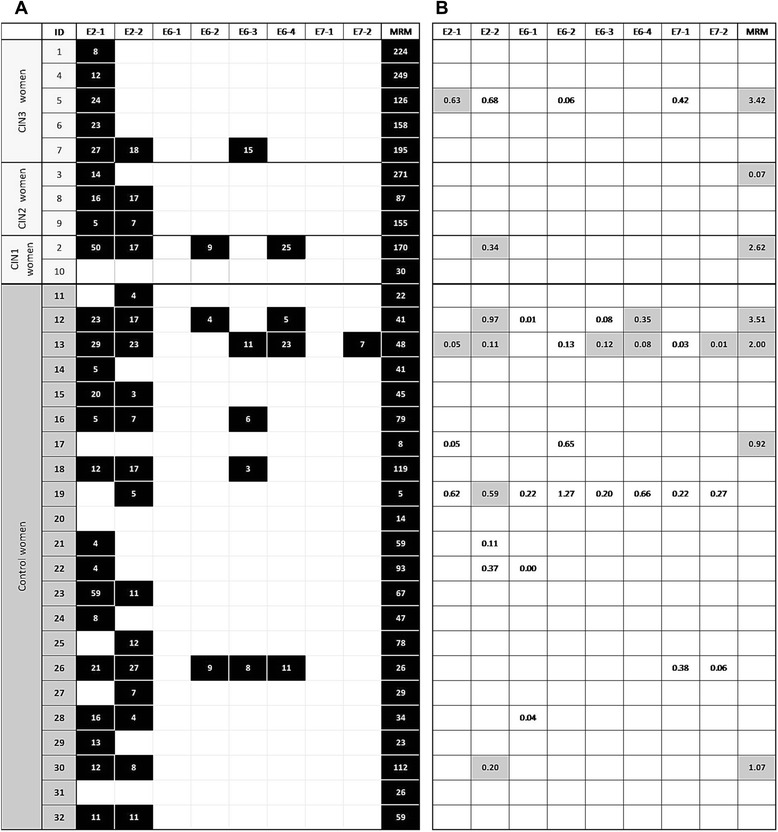
Results from LST and Foxp3 assays. **A)** HPV16 specific LST results among the case and the control groups. Only positive responses are marked as black boxes with stimulation index values under the corresponding peptide pools. Memory response mix (MRM) was used as a positive control. **B)** HPV16 E6 and E7 induced alteration in CD4+ CD25+ Foxp3+ -cell population given as percentages. Only positive (up-regulation) responses are shown. Up-regulation of Foxp3+ cells is defined as at least twice the percentages of that in the medium-only control. Grey box indicates simultaneous positive response in LST.

T-cell reactivity against HPV16 E6 was rare in both groups; no one had any T-cell response to peptide pool E6.1, and a response against E6.2, E6.3 and/or E6.4 was found only in two of the women with incident CIN (ID2 and ID7) (Figures 1 and 2A). Reactivity against HPV16 E6.2, E6.3 and/or E6.4 peptide pools was seen in five controls (ID12, ID13, ID16, ID18 and ID26) (Figures 1 and 2A). Taken together, there was also no difference in T-cell reactivity against HPV16 E6.2-4 peptide pools between the cases (2/10, 20%) and controls (5/22, 23%). T-cell reactivity against E7, either the pool 1 or the pool 2, was not found in any of the CIN women, while one control women had a response against HPV16 E7.2 (ID 13). One woman with CIN1 (ID10) and 3 controls (ID17, ID20, ID31) had no T-cell specific immunity against HPV16. Despite of the lack of HPV16 T-cell mediated immunity, ID10 tested HPV16- seropositive, but only once at 12-month-visit. She had a persistent HPV16 infection in the cervix. Woman ID 17 was HPV16 seropositive at 12 and 24 month-visits although she had no HPV16 -specific CMI (Additional file 1: Table S1).

An interesting observation was also the T-cell reactivity (LST) against the memory response mix (MRM), which was statistically significantly higher among the cases (166.41; median 163.82) than in the controls (48.88; median 43.00) (p = 0.001) (Figure 1). The MRM response of the CIN women was nearly four-fold higher than in the controls.

### Cytokine production by proliferative T-cells

**Figure 3:**
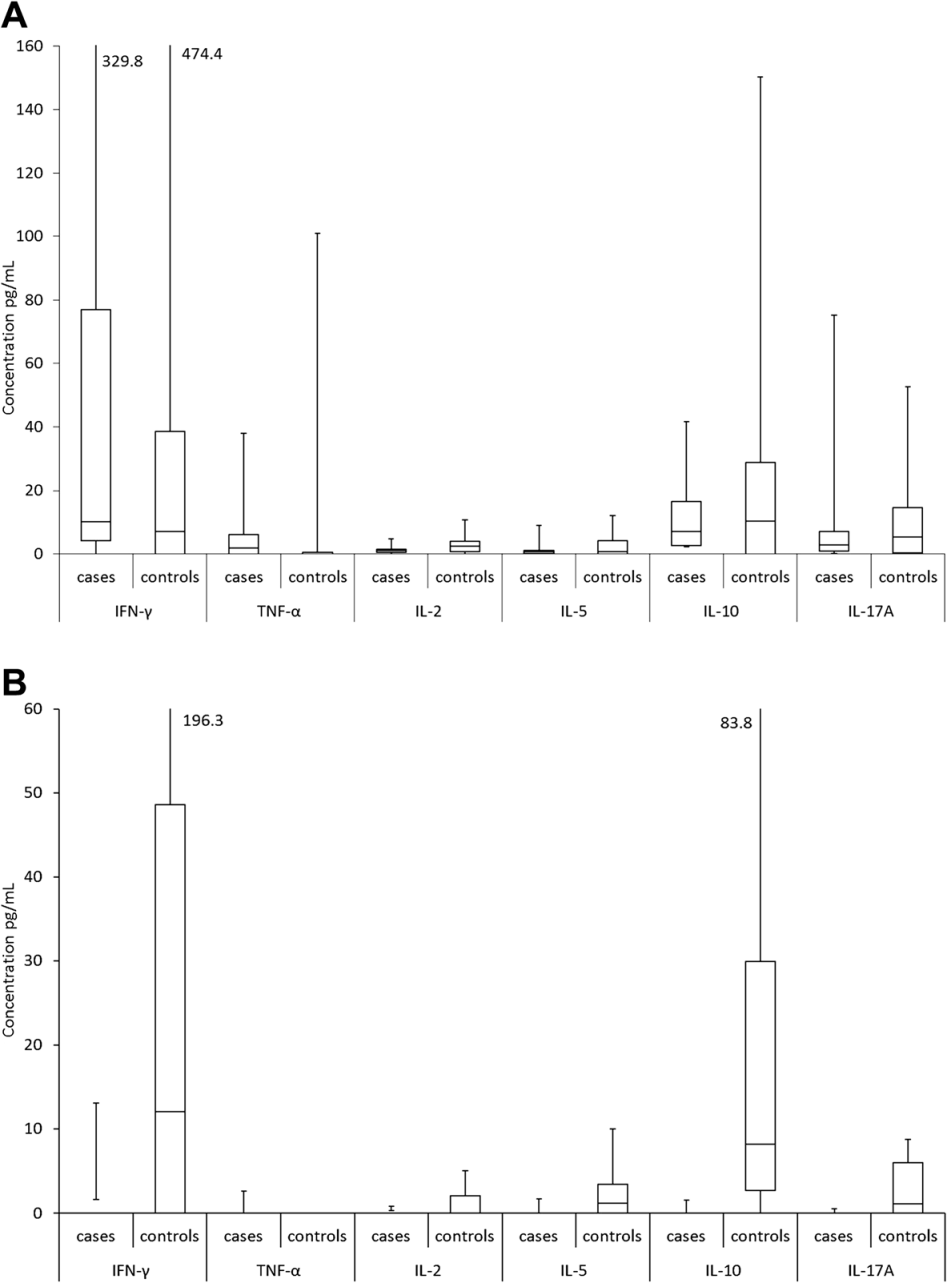
HPV16 E2, E6 and E7 specific cytokine secretions in cases and controls Peptide-specific values are pooled together and the final value is the mean of each pool. **A)** HPV16 E2, **B**) HPV16 E6. Only IL-17A showed statistically significant (p = 0.035) difference between the cases and controls when stimulated with HPV16 E6-specific peptides.

Cytokine secretion after HPV16 E2 and E6 stimulation is summarized in the Figure 3 A and B. As only one woman (ID13) had HPV16 E7 specific LST response, the HPV16 E7 data was excluded from the Figure 3 A and B. In the CIN women, IL-10 was the most frequently secreted cytokine, being detected in all 10 women, followed by IL-17A and IFN-γ, with 90% (9/10), and by IL-2 and IL-5, secreted in 80% (8/10) of the women. TNF-α was the most rarely detected cytokine in the CIN patients, 60% (6/10). In the controls, IL-17A was the most frequent cytokine, found in 95% (21/22) of the women, followed by IL-10 and IL-5 in 86% (19/22) of the controls. IL-2 secretion was found in 77% (17/22), IFN-γ in 68% (15/22) and TNF-α in 64% (14/22) of the controls.

Moreover, HPV16 E2-specific immunity resulted in higher cytokine secretion than HPV16 E6 or E7 both in the cases and in controls. The positive proliferative T-cell immunity was associated with the production of IFN-γ and detected in 80% (8/10) of the CIN cases, mostly due to the pooled effects of HPV16 E2.1 and E2.2 as also the case with IL-10 and IL-17A secretions.

Figure 3 summarizes the cytokine secretion by HPV16 E2 and HPV16 E6 in LST positive wells. No significant differences were found between the controls and the CIN women except for IL-17A. IL-17A levels were statistically significantly higher in the controls (p = 0.035) after HPV16 E6 peptide stimulation than in the CIN women (Figure 3B).

### HPV16 -specific CD4+ CD25+ Foxp3+ regulatory T-cell detection

HPV16 -specific CD4+ CD25+ Foxp3+ regulatory T-cells were detected only at low levels in both the cases and the controls (Figure 2B). Two CIN women (2/10; 20%) and eight controls (8/22; 36%) had Foxp3+ regulatory cell responses to some HPV16 peptide pools.

### Questionnaire about the risk factors of HPV infections

Based on questionnaire, 70% (7/10) of the CIN women and 38.1% of controls analysis (8/21, data missing from one woman) were smokers. Previous genital warts were recorded in 10% (1/10) of the cases and in 40% (8/20) of the controls, but the difference was not statistically significant. No significant differences were seen either in medication, number of sex partners, different sexual habits, alcohol consumption and STDs between these two groups.

**Figure 4:**
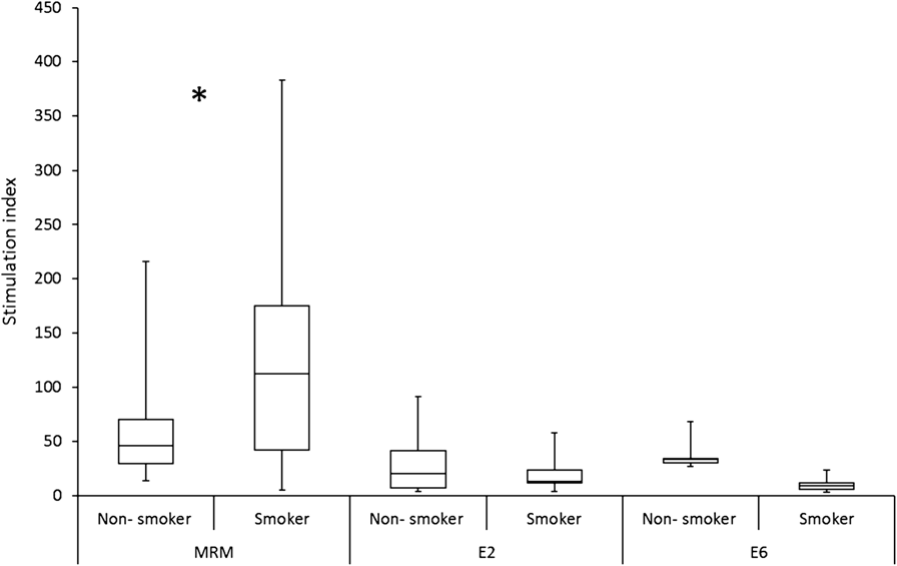
The LST responses related to smoking in women. The stimulation indexes of peptide pools of HPV16 E2 and E6 are combined. Only one non-smoking woman responded to one pool of E7 peptides. *Non-smoking women had statistically lower LST MRM response than the smokers (p = 0.032).

We also analyzed the LST response to HPV16 peptides and MRM stratified by smoking, use of alcohol, genital warts, oral sex habits and number of sex partners. Smokers and users of alcohol had significantly higher LST MRM response than the non-smokers or non-drinkers (p = 0.032 and 0.045, respectively), but no differences were found in CMI responses against HPV16 E2, E6 and E7 peptides (Figure 4). Women with previous genital warts had a tendency to higher response to HPV16 E6-4 peptides than women without the warts (p = 0.054). Women with higher numbers (>10) of previous sex partners had higher response to HPV16 E6-3 than women with fewer sex partners (p = 0.047).

Based on oral HPV and HPV serostatus, women were classified into four groups as followes: Group 1, women with cervical HPV16 and a CIN lesion caused by HPV16 (n = 5); Group 2, women with cervical HPV16, but her CIN lesion was associated with other or unknown HPV genotype (n = 5); Group 3, women with oral HPV16 and HPV16 -specific antibodies, but no cervical HPV infection (n = 14); Group 4, women with oral HPV16, but neither HPV16-specific antibodies nor cervical HPV16 infection (n = 8). The results of the LST and cytokine analyses stratified by the groups are shown in Table 1. No significant differences were seen between these four groups when lymphocytes were stimulated by HPV16-specific E2 or E7 peptide pools. When stimulated by MRM, the lymphocytes of the women in group 1 secreted significantly higher amount of IFN-γ level than the other groups. Also in LST the MRM stimulation produced significantly higher SI values in group 1, than in groups 3 and 4 (p = 0.023 and p = 0.021) while the highest SI values were found in group 2 (p = 0.0001). The group 4 had highest IFN-γ secretion levels when stimulated by HPV16 E6.4 peptide pool (p = 0.034) and also the highest IFN-γ levels when the response of all HPV16 E6 peptide pools were pooled together (p = 0.050) (Table 1).

**Table 1:** Cytokine and LST results of the women in the four groups with different HPV-status

**Cytokine results**					
**Stimulant**	**Cytokine**	**Study groups**	**Mean value I**	**Mean value II**	**p value**
MRM	IFN-γ	Group 1 vs. Group 2	**908.087**	309.916	0.026
		Group 1 vs. Group 3	**908.088**	275.220	0.003
		Group 1 vs. Group 4	**908.089**	404.942	0.043
HPV16 E6-4	IFN-γ	Group 3 vs. Group 4	5.220	**56.783**	0.034
HPV16 pooled E6	IFN-γ	Group 3 vs. Group 4	11.318	**69.173**	0.050
**LST results**					
	**Stimulant**	**Study groups**	**Mean value I**	**Mean value II**	**p value**
	MRM	Group 1 vs. Group 2	118.493	**214.323**	0.005
		Group 1 vs. Group 3	**118.493**	51.455	0.023
		Group 1 vs. Group 4	**118.493**	44.385	0.021
		Group 2 vs. Group 3	**214.323**	51.455	0.0001
		Group 2 vs. Group 4	**214.323**	44.385	0.0001

Group 1 (n = 5): HPV16 + and a CIN lesion caused by HPV16; Group 2 (n = 5): HPV16 + and a CIN lesion with another or unknown HPV genotype; Group 3 (n = 14): Oral HPV16+/HPV seropositive and no cervical HPV; Group 4 (n = 8): Oral HPV16+/HPV seronegative and no cervical HPV.

## Discussion

In the present study, we have assessed the HPV16-specific T-cell immunity in the women who developed HPV16-associated CIN during the FU. The results were compared with those of the control women, who tested constantly HPV-negative in their genital samples. Although the number of women was limited, the main strength of this study is the long FU time with detailed data on HPV DNA status, not only of the genital tract but also of oral mucosa. In fact, this is the first study were also the FU data on oral HPV status is reported together with the genital HPV data.

To our surprise, the HPV16-specific CMI against E2-, E6- and E7 peptide pools was similar among the women who developed CIN and in those who tested constantly HPV-negative. The FU data showed that the cases and the controls were similar with regard to the presence of either oral HPV16 DNA or HPV16 antibodies in sera. Our results indicate also that these healthy women have been exposed to HPV earlier in their life but the infection has either totally resolved or has never truly developed. Thus, the T -cell responses reported here could be induced by any HPV infection, either resolved or still present, in any site of the body. However, one would expect to find some differences in HPV-specific immunity in the women who developed severe HPV-induced cervical disease as compared to those who remained completely HPV-negative. For example, we recently demonstrated that the clearance of oral high-risk HPV infection is impaired by long-term persistence (24 M+) of cervical HPV infection [32].

In the present study, we also found one interesting association between oral HPV and CMI after re-grouping the women into four groups on the basis of their HPV type in the CIN lesion, HPV serology and oral HPV status. The women with oral HPV16 DNA, but testing HPV-seronegative and being also HPV DNA-negative during the FU (Group 4), had the highest IFN-γ secretion level after stimulation with HPV16 E6.4 peptide pool. One could speculate that productive oral or oropharyngeal HPV infection with E6 expression could result in the local cell mediated mucosal immunity, which also protects from cervical HPV infection. Oral administration of lactobacilli bearing the surface-displayed E6 protein has been shown to induce T cell-mediated cellular immunity and antitumor effects in mice [33].

Importantly, our results also indicated that oral mucosa and its HPV infection as such could not explain the similarity of HPV16-specific CMI in women with and without CIN. Unfortunately, we did not have data on anal HPV status to get a more comprehensive overview on HPV-induced immunity as related to the presence or absence of HPV DNA.

Of note is the fact that all patients were treated for their CIN before entry in this CMI study, and their blood samples have been taken after (31-129 months later) diagnosis of the cervical disease. Thus, HPV16-specific CMI might have changed during the FU after CIN treatment. One would even expect to see a better HPV-specific CMI in these women after cone treatment. However, we found no evidence of impaired or improved HPV-specific CMI in these women as compared with HPV-negative controls. In fact, it has been shown before that the number of HPV16 E2 specific T-cells are high regardless the outcome of HPV infection after treatment [17]. Alternatively, the E2 response could reflect some degree of cross-reactivity to other HPV types as has been reported earlier [13]. Instead, the lack of CMI reactivity to HPV16 E6 and E7 peptides could indicate a long-term absence of HPV16 virus in these women.

Another observation of interest was that HPV16 E6 stimulation resulted in significantly higher secretion of IL-17A by T-cells in the controls than in women with CIN (p = 0.035). IL-17A has a key role in host defense against infections, and the pathogenesis of some autoimmune and chronic inflammatory diseases. The role of IL-17A in cancer development is controversial [34]. High proportion of IL-17A producing CD4+ T-cells in women with CIN have been reported [35]. Contradictory to that, women with LSIL showed higher IL-17A levels than women with high-grade SIL [36]. Recently, Gosmann and coworkers demonstrated an immunosuppressive role for IL-17 in HPV-associated epithelial hyperplasia and suggested that blocking IL-17 in persistent viral infection may promote antiviral immunity and prevent progression to cancer. IL-17A can be produced by Tregs as well as by Th17 cells. The latter also produce other cytokines including IFN-γ. Whereas IL-17A with Tregs is associated with more severe disease, Th17 cells (producing IL17 and IFN-γ) seem to be protective [37]. One can speculate that the segregation of IL-17A is changed at some stage of viral infection and tumor development. Our results presented here show that the control women had both higher levels of IL-17A and IFN-γ in LST+ samples and also in HPV16 peptide-specific responses than the cases, indicating that Th17 might play an important protective role in HPV induced cervical diseases.

We also found that there is no HPV16 E6.1 specific CMI either in CIN patients or in controls, indicating that this site is not relevant for HPV-induced immunity. As far as we are aware, there are no other studies reporting the CMI response against HPV16 E6.1 peptide pool (amino acid 1-50).

Interestingly, the CIN women had T-cell hyperactivity against the common antigens as indicated by the nearly four-fold higher MRM response than in the controls (p = 0.001). One explanation for their higher MRM response in the CIN women might be *Candida Albicans* as one of the antigens present in MRM. *C. albicans* as a component of the normal flora often colonizes the skin and mucosal surfaces of healthy individuals. Underlying acquired immunity to *C. albicans* is usually present in immunocompetent individuals [38]. *C. albicans* is capable of triggering an expansion of specific or naïve T-cells [39,40]. Candida infection is very rare in CIN or even in warty HPV-induced lesions. Furthermore, studies on cytological smears are also in line with these observations [39]. Murta and coworkers (2000) showed that *Gardnerella vaginalis* was the most frequent agent in women with HPV infection (23.6% versus 17.4%; P <0.05), while in the control group the most frequent agent was *Candida* (23.9% versus 13.8%; p <0.001). Accordingly, one would expect to find higher MRM response in the controls than in CIN patients. However, slightly higher MRM levels have been reported among the controls than in women with cervical cancer (CC) [10,41]. Our results also indicate that the high MRM response was related indirectly rather to the presence of genital HPV and not oral HPV. The mechanism remains obscure and need further studies.

We also compared the demographic data with the HPV-specific immunity. Atopic symptoms in childhood have been associated with an increased risk to acquire CC later in life [42,43]. In the present study, atopic symptoms recorded by questionnaire were equally rare in both groups: 10% in the CIN group and 5.3% among the control women. Women with higher numbers (>10) of previous sex partners had higher response to HPV16 E6-3 than women with fewer sex partners (p = 0.047). This might indicate higher exposure of women with multiple partners to HPV16 but also to different variants of HPV16.

Previous studies have shown that smoking increases the risk for CC. Several studies have confirmed that smoking increases the risk to contract HR-HPV infections and their persistence [24,44,45] and the positive association between HPV infection, smoking and CC is well documented in many studies [38,46-48]. Our previous results from this same cohort have shown that smoking also increases the risk for persistent genital and oral HPV infection [4,5,32]. This might be explained by an impaired immune response caused by smoking, or smoking might aid HPV infection to become persistent by an unknown mechanism not related to immunity. In this analysis, however, HPV16 specific CMI was similar in smokers and non-smokers but response to MRM was improved in smokers, suggesting that smoking can affect CMI. Smoking is known to increase the risk for oral candidiasis which could explained the high response to MRM in these women.

## Conclusions

To conclude, our results show that there are no differences in the HPV16-specific CMI except the higher secretion of IL-17A by the HPV16 E6 in the controls. Interestingly, the CIN women were also hyper-reactive against common antigens which prompt further studies. Smoking can stimulate the reactivity to common antigens but had no direct effect on HPV-specific CMI.

## Additional file

Additional file 1: Table S1. Characteristics of the women with CIN (cases) and HPV-negative women (controls) according to HPV status, cervical disease and HPV serology during the FU. ND = no data, T = time of treatment of CIN, D = time-point of CIN diagnosis, NA = not applicable, * = time of blood sample from the diagnosis of CIN.

## Abbreviations

CBA: Cytometric bead array
CC: Cervical cancer
CIN: Cervical intraepithelial neoplasia
CMI: Cell-mediated immune
CPM: Counts per minute
Foxp3: Forkhead box P3
FU: Follow up
HPV: Human papillomavirus
HR: High-risk
LSIL: Low-grade squamous intraepithelial neoplasia
LST: Lymphocyte stimulation test
MRM: Memory response mix
SI: Stimulation index
SPPS: Solid phase peptide synthesis
